# Current State and Perspectives of Simulation and Modeling of Aliphatic Isocyanates and Polyisocyanates

**DOI:** 10.3390/polym14091642

**Published:** 2022-04-19

**Authors:** Veniero Lenzi, Anna Crema, Sergey Pyrlin, Luís Marques

**Affiliations:** Physics Center of Minho and Porto Universities (CF-UM-UP), University of Minho, Campus de Gualtar, 4710-057 Braga, Portugal; annapaulacrema@gmail.com (A.C.); pyrlinsv@fisica.uminho.pt (S.P.); lsam@fisica.uminho.pt (L.M.)

**Keywords:** aliphatic isocyanates, atomistic modeling, molecular dynamics, coarse-grained models

## Abstract

Aliphatic isocyanates and polyisocyanates are central molecules in the fabrication of polyurethanes, coatings, and adhesives and, due to their excellent mechanical and stability properties, are continuously investigated in advanced applications; however, despite the growing interest in isocyanate-based systems, atomistic simulations on them have been limited by the lack of accurate parametrizations for these molecular species. In this review, we will first provide an overview of current research on isocyanate systems to highlight their most promising applications, especially in fields far from their typical usage, and to justify the need for further modeling works. Next, we will discuss the state of their modeling, from first-principle studies to atomistic molecular dynamics simulations and coarse-grained approaches, highlighting the recent advances in atomistic modeling. Finally, the most promising lines of research in the modeling of isocyanates are discussed in light of the possibilities opened by novel approaches, such as machine learning.

## 1. Introduction

Isocyanates are a well-known family of versatile molecules, first synthesized in the middle of the 19th century by C. A. Wurtz [1]. Many years later, in 1932, Otto Bayer discovered a method to produce polyurethanes (PU) [2], which uses isocyanates as a basic building block. Since then, they have rapidly become one of the most important molecules in industrial chemistry, due to the extreme versatility and diffusion of polyurethane materials [3,4]. The importance of isocyanates is not restricted to the vast field of PU chemistry, but they are a key component for the synthesis and production of advanced materials. Moreover, isocyanates became relevant molecules in astrochemistry and astrophysics, following their discovery in the interstellar medium [5].

In the large family of isocyanates, aliphatic polyisocyanate trimers and higher-order polymers are particularly interesting, since they combine strong mechanical strength and chemical stability to crosslinking capabilities and biocompatibility, hence rendering them very appealing in the design and synthesis of novel PU-based materials. 

Despite the avenues opened by experimental research efforts on aliphatic isocyanates, the simulation community lagged behind, with the results that the availability of accurate atomistic models capable of reliable predictions of the physical properties of isocyanates and polyisocyanates is limited, especially when coming to fully atomistic molecular dynamics simulations. This means that the possibility to explore and understand through simulation and modeling the complex interplays between chemical composition and reactivity, local structure, and physical properties of isocyanate-based materials is limited.

Given the above, the scope of this review is to give an overview of the active research fields on isocyanates, in order to demonstrate their centrality in contemporary research and the underline need for accurate models. In this optic, we will provide a summary of the atomistic simulation works regarding isocyanates, with a focus on recent achievements in the field of atomistic modeling and the possibilities offered by novel techniques, such as machine learning. 

## 2. Isocyanates and Polyisocyanates: Definitions and Chemistry

**Figure 1:**
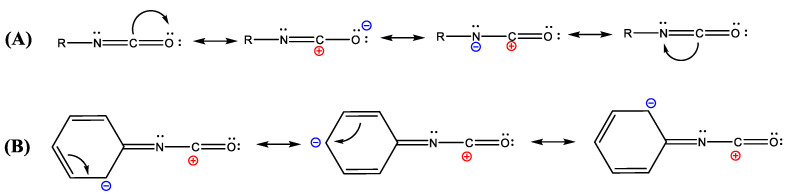
Hybrid resonance structures of the isocyanate functional groups for aliphatic (**A**) and aromatic (**B**) structures.

Before discussing the applications of isocyanates, it is useful to introduce the chemistry of isocyanates and their nomenclature. The isocyanate group chemical structure is made by two adjacent double bonds connecting nitrogen (N), carbon (C), and oxygen (O) atoms, as shown in Figure 1A. The peculiar position of the C atom, between the two electronegative atoms N and O, induces a relatively large positive charge on it, resulting in a strong electrophilic effect. Thus, the nucleophilic character of the N atom coupled with the high electrophilicity of the C atom is what makes isocyanates so unique and widely employed. 

Isocyanates are divided into aromatic and aliphatic structures. They are applied differently and are rarely interchangeable. As the name indicates, aromatic isocyanates have an aromatic moiety in the radical group, such as toluene diisocyanate (TDI), methylene diphenyl diisocyanate (MDI) or xylene diisocyanate (XDI), while aliphatic ones contain only alkyl chains, such as the hexamethylene diisocyanate (HDI) and isophorone diisocyanate (IPDI). 

When compared to aliphatic ones, aromatic isocyanates are characterized by a higher reactivity with active hydrogens, due to the negative charge delocalization which is stabilized by the resonance effect of the aromatic ring (Figure 1B). 

While the highest reactivity of aromatic isocyanates is an advantage in terms of reaction and conversion rates, conversely, the lack of selectivity and consequently reaction control is certainly a disadvantage. The presence of substituents also affects the reactivity of the isocyanate group, with electron-withdrawing substituents in ortho or para position increasing it while donating substituents reducing it [6]; moreover, PUs based on aromatic isocyanates typically have less resistance to sunlight and UV exposure, weak gloss retention and significant discoloration when compared to aliphatic isocyanates. 

**Figure 2:**
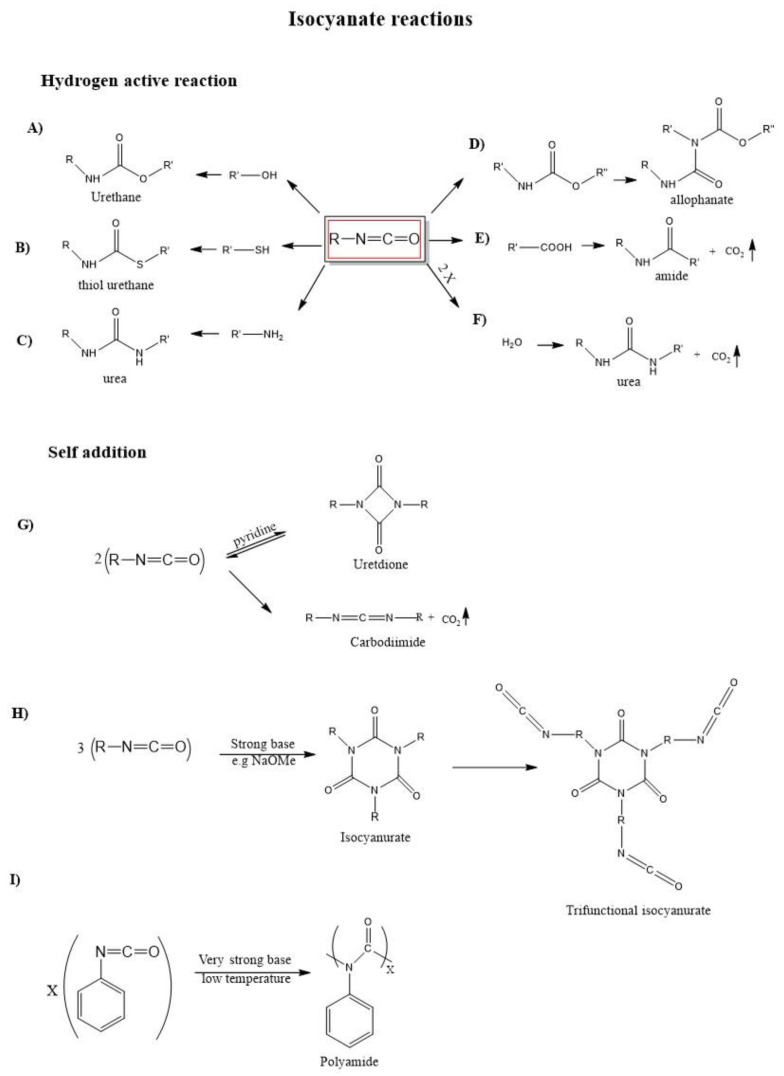
Main isocyanates reactions and their products. Schemes (**A**–**F**) represent reactions with an active hydrogen-containing group, while schemes (**G**–**I**) represent self-addition reactions. In all schemes R = −(CH_2_)n−.

Figure 2 shows the typical-NCO group reactions and their products [7]; they are divided into two classes: addition reactions (primary and secondary) with an active hydrogen-containing compound, such as amine, water, alcohol, carboxylic acid, urethanes (Figure 2A–F) and self-addition reactions (Figure 2G–I). Urethane formation (A) occurs when an isocyanate reacts with an alcohol group, forming a urethane linkage, or with a sulfur group (B), to form a thiourethane molecule. Urea is obtained when isocyanate reacts with amine groups (C) or water (F), with the release of gaseous carbon dioxide. The release of CO_2_ is present in some reactions helping the formation of PU foams. The reaction between isocyanate and carboxylic acid (E) gives amide product, and finally, in (D) isocyanate reacts with urethane giving an allophanate molecule.

The dimerization reaction (Figure 2G) of isocyanates results in the reversible formation of a difunctional uretidione. Cyclotrimerization reaction of linear diisocyanates results in the formation of a ring structure called an isocyanurate (Figure 2H) which, unlike the uretdione, is very stable. Although quite slow if uncatalyzed [4], the dimerization and cyclotrimerization reactions are spontaneous and, because of this, they limit the shelf-life of isocyanate products [7]. 

The functionality “ν” is defined as the number of unreacted NCO groups in an isocyanate molecule. Combining three linear diisocyanates will result in a trifunctional isocyanurate. Clearly, isocyanurates can be further combined to obtain molecules with higher functionality. Conversely, combining three linear mono-isocyanates will result in nonfunctional isocyanurates, which are equivalent to Figure 2H, except that the terminal NCO groups of alkyl arms are substituted by −CH_3_ terminations.

## 3. Applications and Research Trends

**Figure 3:**
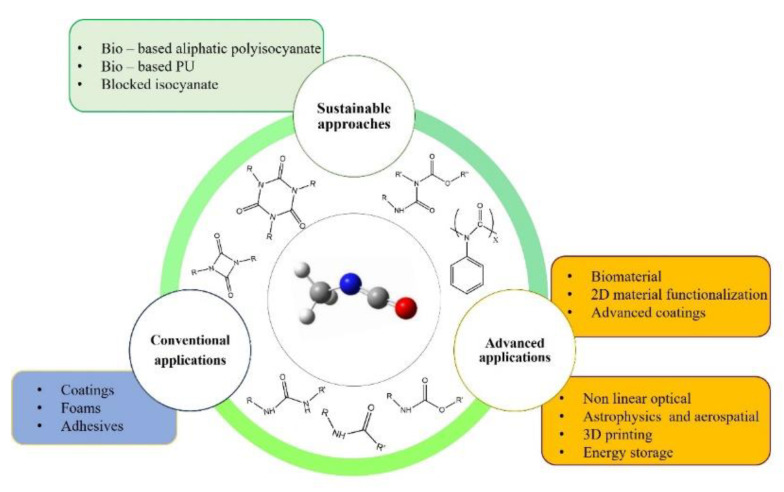
Overview of the applications of isocyanates discussed in Section 3.

Isocyanates are very versatile molecules and nowadays are employed in many different fields, as summarized in Figure 3. 

The most important application of isocyanates is in polyurethane (PU) production, of which they are one of the precursors, along with polyols. According to data on compounds [8], the annual growth PU materials market was estimated at USD 18 billion in 2020 with an increase of 5–6% in the period 2021–2025. 

The incredible versatility of PU materials and their properties makes them ubiquitous: they are used as construction blocks, insulating foams, and flame retardants in the building industry, coatings in the automotive and space industry, and certain compositions are suitable for the use as biomaterials [3,4,9]. 

When aliphatic isocyanates and isocyanurates are employed, materials with better properties are generally obtained, when compared with other families. This is due to the fact that, when used, tri- and polyfunctional aliphatic isocyanurates combine crosslinking capability with the excellent mechanical properties, degradation resistance, and chemical stability provided by the isocyanurate ring [10,11,12,13,14]. Because of this, they are the basis of high-performance coatings [15,16,17] and porous materials [18,19], while their good optical properties make them ideal to be used as a matrix for nonlinear optically active devices [20]. 

**Figure 4:**
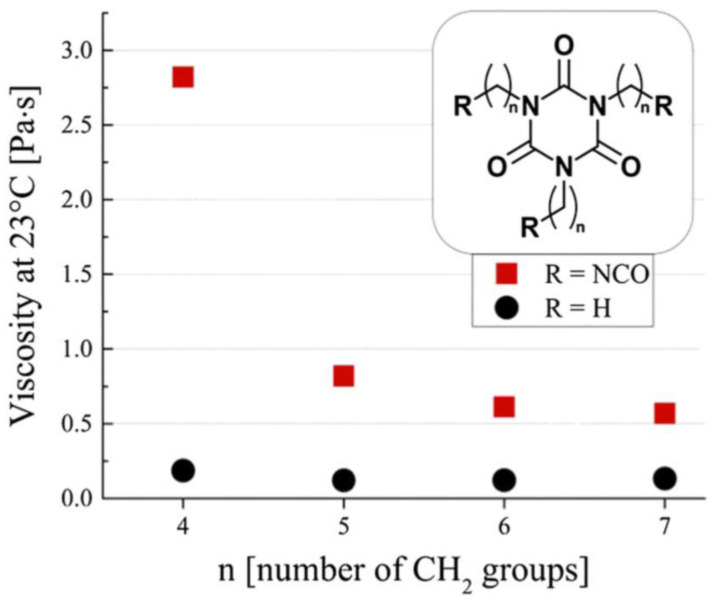
Measured viscosity for functional(squares) and nonfunctional(circles) isocyanate trimer pure liquids, expressed as a function of the alkyl arm length. Reproduced from Ref. [22].

However, the viscosity of polyisocyanates grows with the degree of polymerization, constituting a limiting factor in their application [21]. A higher viscosity directly translates to difficulties in the application and mixing of the polyisocyanate precursor melt, and for this reason, lower-viscosity isocyanurates are mostly desired, possibly retaining their functionality. In this regard, the synthesis of ultra-pure functional and nonfunctional isocyanate trimer liquids was conducted, and their viscosity was measured [22]. As shown in Figure 4, it was revealed that, in the case of functional trimers, the viscosity is strongly influenced by the alkyl arm length, with higher viscosities observed for lighter functional trimers (i.e., with shorter alkyl arms), while no such dependency and a much lower overall viscosity was found in the case of nonfunctional trimers. 

The need of understanding this peculiar behavior of isocyanurate liquids led to the first efforts in careful atomistic modeling and simulation of the liquid phase of aliphatic polyisocyanates.

PU-based microgels, that is polymer networks of micrometer-scale dimension [23], have been synthesized and used for several biomedical applications, such as targeted drug delivery [24,25,26], vascular embolization [27], shape memory biomaterials with improved lifetimes [28]. Isocyanurate-crosslinked hydrogels are a very interesting class of materials, due to their intrinsic versatility [29] and biocompatibility [9]. It has been shown that by using isocyanate-end functionalized polymers, hydrogels with very good control of the internal structure can be obtained [30,31], overcoming the limitations of traditional crosslinking techniques, where an already formed crosslinker is added to the precursor melt; such hydrogels show improved mechanical properties in the swollen state and have shown promising potential in the production of contact lenses [32]. 

As already highlighted, the NCO group is very reactive. It is then not surprising that isocyanates allow for a quick route for surface functionalization. In these applications, the NCO group of an isocyanate-functionalized polymer reacts with the surface, forming covalent bonds and resulting in polymer grafting. In this way, fluorinated polymers were grafted to glass, providing it with antifogging and self-cleaning capabilities [33], while other substrates, such as nanocarriers [34] and cellulose [35] have been functionalized in this way. The same mechanism can also be used for the immobilization of various species on a substrate [36,37]. Graphene is no exception: isocyanate functionalization allows for an easier exfoliation and solubility of graphene oxide sheets [38] while making them embeddable in a matrix to exploit the unique graphene properties [39,40,41,42]. 

In coatings technology, a way to improve the coating lifetime is by providing it with self-healing capabilities. In this context, aliphatic [43] and non-aliphatic isocyanates [44,45,46] have been used in the form of microcapsules. In these microstructures, the reactive isocyanate molecules are stored within the capsule and are released upon damage. This allows the isocyanates to react with the coating and establish new linkages, effectively restoring the coating’s integrity; the same philosophy has been adopted in wood adhesives [47]. In general, with the appearance of new synthesis routes [30,48,49] and novel processing and handling techniques, such as molecular layer deposition [50], the role of aliphatic isocyanates in the production of advanced materials is expected to grow. For instance, the possibility of employing isocyanates in reactive inkjet 3D printing processes is very appealing [51]. 

Owing to the priority of finding sustainable and environmentally friendly processes in chemistry, alternatives to the traditional synthesis methods are thoroughly investigated, aiming at finding petrol-free sources for the reactants and at reducing water consumption and polluting compounds’ emissions. In this light, aliphatic isocyanates could be obtained through green chemistry routes [11] and in 2015 the first commercial bio-based aliphatic polyisocyanate product was introduced into the market [52]. These could be combined with bio-based PU formulations [53]. The increased mechanical strength of aliphatic isocyanate trimers allows for the use of starch as polyol [10]. In fact, the use of starch in the production of PU composites not only yields but makes the final PU products biodegradable, although the hydrophilic nature of starch limits its dispersion in hydrophobic PU polymers [54].

Blocked isocyanates [4] are also a promising way to provide a longer shelf-life and lower toxicity than free isocyanates. The blocking reaction of the isocyanate group with the active hydrogen of the blocking agent makes the NCO group non-reactive since the weak bond between the active hydrogen and the N atom produces a compound that is inert at room temperature but reactive at high temperatures. Furthermore, the use of blocked isocyanates allowed for a safer and more controlled synthesis of urethanes. As an example, Rolph et al. [55] synthesized a novel monomer, methacryloyl pyrazole, based upon blocked diisocyanates to produce a crosslinked polymeric particle by the postpolymerization reaction. This might allow a wide range of applications, such as thermally responsive latent catalytic systems, formation of complex molecular architectures, and single-chain polymer nanoparticles. 

Another way to improve the isocyanate production process from a green chemistry perspective is through the development of novel catalysts which could provide more efficient production routes under milder conditions. In this sense, many promising options have been explored [12,13,14,56,57,58].

In 2014, the Philae lander of the Rosetta spacecraft landed on the comet 67P/Churyumov–Gerasimenko and analyzed its composition, finding methyl isocyanate [5]. This confirmed the existence of isocyanates in the extraterrestrial space and, following this discovery, methyl isocyanate and possibly ethyl isocyanate were observed in interstellar medium and protostar gas clouds [59], identified by the strong and peculiar spectral line of the NCO group. The presence of the ethyl isocyanate in the interstellar medium has been later confirmed [60]. This claim is further supported by recent studies addressing the formation and stability of isocyanates under astrophysical conditions [61,62], indicating that methyl isocyanate/water mixtures appear to be stable at temperatures below 20 K. In fact, elementary isocyanates are one of the very basic ingredients of life, being amino acid precursors [63], thus understanding the behavior of isocyanates in these extreme environments allows us to shed light on the chemical evolution that from elementary molecules led to those capable of sustaining life [64].

## 4. Modeling and Simulation of Isocyanates

Having established the centrality and importance of isocyanates, not only in polyurethane research but also in other fields, we will now focus on reviewing the theoretical and computational studies on them. In Table 1, we summarize the main advantages, drawbacks, and application fields of the methods treated in this section.

### 4.1. Ab Initio Studies

In view of the discussion on atomistic models for aliphatic isocyanates, it is important to consider the more fundamental first-principles studies, since in many cases they offer the only way to obtain reference data upon which to build classical atomistic models. 

First-principles, or *ab initio*, methods have been historically focused on understanding the reactivity and reaction pathways of isocyanates, while a lower consideration was given to their physical properties and intermolecular interaction in gas and condensed phases. Moreover, most studies address very simple isocyanates, such as isocyanate ion (NCO-) or isocyanic acid (HNCO), while a lower number target more complex ones. This is even more true for aliphatic isocyanates. As a consequence of this, the liquid phase properties of isocyanates have not been characterized from the *ab initio* perspective as well as their chemistry, and this scarcity is directly reflected by the reduced availability of classical atomistic models. 

Isocyanates-containing species appear as intermediate compounds in catalytic processes on metallic surfaces involving carbon monoxide and nitrogen oxides [65]. For this reason, first-principle studies employing density functional theory (DFT) [66,67] have been conducted in order to understand the interactions between the isocyanates and the catalytic surfaces. Among the considered metals, there is copper [68,69,70], silver [71], palladium [72], aluminum oxide [73], gold [74]. In all these cases, strong chemisorption between the isocyanate species and the substrate was found, with the formation of a bond involving the N atom. Similar studies have been conducted regarding the adsorption of isocyanic acid on titanium dioxide and aluminum oxide surfaces [75,76], because of its importance in the selective catalytic reduction processes of diesel engine exhausts. 

Isocyanic acid, whose crystal structure resembles the one of carbon monoxide [77], is also supposed to play a role in the chemistry of interstellar ices and comets [78], therefore computational *ab initio* studies tried to understand its behavior in the extreme space conditions. In this sense, DFT has been used to calculate the vibrational spectrum of methyl isocyanates, in order to relate it with the astrophysical observations by Maté and collaborators [61]. Incidentally, they used isocyanic acid as a starting point to guess the methyl isocyanate crystal structure. Stable geometries of isocyanic acid-water clusters, now expected to be found in astrophysical environments, have been studied with *ab initio* methods [79,80]. More recently, *ab initio* molecular dynamics coupled with metadynamics demonstrated that the presence of water and water/CO ice actually helps the formation of methyl isocyanate [81].

Moving the attention to aliphatic isocyanates and isocyanurates, the cyclotrimerization reaction has been thoroughly investigated using *ab initio* method. Okumoto and Yamabe [82] firstly studied the ring formation using DFT, focusing on the effect of the presence of a catalyst and suggested that catalysts are needed to obtain trimers. A later study [83] indicated that cyclotrimerization can still happen without a catalyst, with an uretdione (i.e., an isocyanate dimer) appearing as an intermediate. Both works agree in suggesting that, when a catalyst is present, cyclotrimerization formation goes through an activated catalyst-isocyanate complex and uretdione appears as an intermediate state, and was further confirmed by different studies [84,85]. This kind of information is extremely useful for the definition of simple reaction models in studies that aim to investigate the crosslinking process during network formation since the final state will be strongly influenced by the reaction features (such as speed and reversibility character). Apart from the trimer formation process, other reactions of aliphatic isocyanates have been investigated as well, such as the urethane bond formation between isocyanates and alcohols [86]. 

Adhesion properties of isocyanates are of great interest because adhesives are one of the main application fields of isocyanates. The adhesion of polyisocyanates on aluminum [87] and steel [88] has been investigated, but these studies were limited to aromatic ones. 

**Figure 5:**
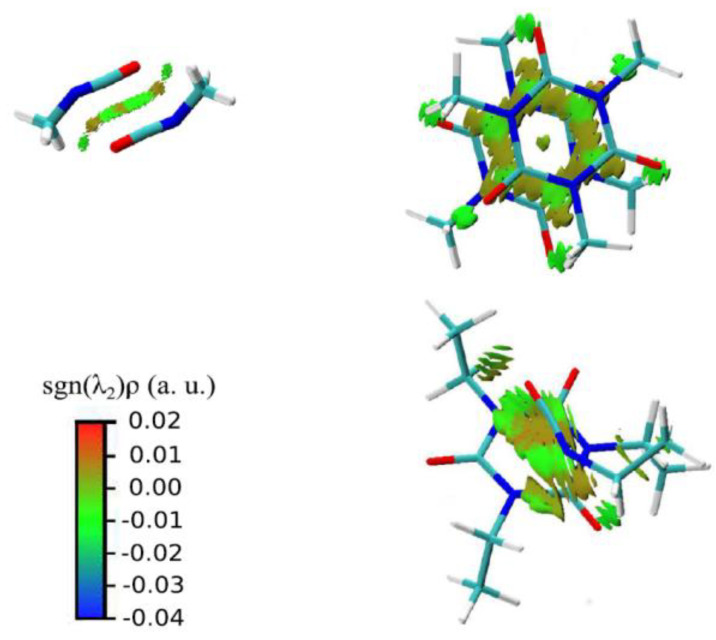
Representation of the intermolecular interaction in aliphatic isocyanurates. From top left and clockwise: NCO–NCO interaction, isocyanurate–isocyanurate interaction, NCO–isocyanurate interactions. The colored regions are the result of an NCI calculation [90]. Reproduced with permission from Ref. [89].

In a more general perspective, the knowledge of the physical origin of molecule-substrate and even molecule-molecule interactions of isocyanates and polyisocyanates can certainly shed light on their peculiar behavior, such as it was observed for viscosity, but can also provide a solid ground for further parametrization and modeling; however, the literature regarding aliphatic polyisocyanates is scarce in this regard. A comprehensive study of the intermolecular interactions of aliphatic isocyanurate has been conducted using *ab initio* calculations and MD simulations [89]. In functional trimer melts, three different interactions were found, as schematically reported in Figure 5, that is the interaction between the NCO groups, one between isocyanurate rings, and one between NCO groups and isocyanurate rings, which was revealed for the first time. Interestingly, all interactions involving isocyanurate rings are dominated by dispersion forces. 

The importance of noncovalent interactions [90] in isocyanate systems has also been confirmed by a study on the stability of diphenyl diisocyanate uretdiones [91], where aromatic *_π_* stacking and van der Waals have been suggested as stabilization mechanisms for the dimer; further, in the case of isocyanuric acid, these interactions are responsible for the stability of complex supramolecular structures, such as rosette motifs [92] or molecular cages [93]. 

### 4.2. Molecular Dynamics Force Fields

Atomistic modeling techniques, namely MD and coarse-grained (CG) models are among the most effective ways with which it is possible to accurately calculate the macroscopic properties of complex polymer systems, such as crosslinked networks and liquid while connecting them with their atomic-scale interactions. 

However, the application of these methods to isocyanates and isocyanate-based systems, such as precursor melts, is hampered by the lack of accurate parametrizations. Isocyanates have not been considered in the initial training set of the major force fields [94,95,96,97], which have been mostly interested in organic compounds of biological interest, and in fact, until recently, there were no specifically parametrized force fields for aliphatic isocyanates and isocyanurates, thus accurate simulations were not possible. To have a quantitative idea, while the experimental viscosity of hexamethylene diisocyanate (HDI) at 293 K is 2.3 mPas, calculating it in MD with an off-the-shelf GAFF [96] force field results in a tenfold overestimation, with a value of 21.7 mPas [98]. More basic properties are overestimated as well by GAFF, with a predicted density and vaporization enthalpy for HDI at 293 K of 1.084 g/cm^3^ and 117.1 kJ/mol, respectively, which compare with the experimental values of 1.053 g/cm^3^ and 67.2 kJ/mol. 

**Figure 6:**
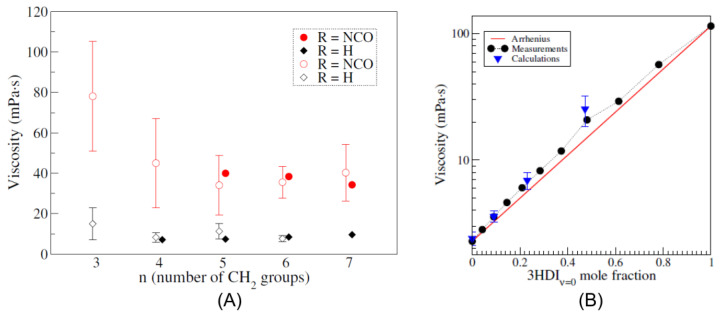
(**A**) Calculated viscosities (open symbols) for functional (circles) and nonfunctional (diamonds) trimer pure liquids at 298 K, as a function of the alkyl arm length, compared with experimental results (full symbols). (**B**) Comparison of the calculated (triangle) and experimental (circles) viscosity of a mixture of hexamethylene diisocyanate (HDI) and a nonfunctional trimer, at 298 K. The red lines indicate the ideal Arrhenius behavior. Reproduced from Ref. [99].

To fill this gap, a parametrization was developed [98] for aliphatic isocyanates and isocyanurates, with the objective to improve the predictions for these molecules and then use MD to investigate the connection between isocyanate molecular structure and viscosity. The parametrized force field was proven to dramatically improve the prediction accuracy of various physical quantities of isocyanates and polyisocyanates liquids. For instance, densities and vaporization enthalpy predictions greatly improved after the reparametrization, with calculated values for HDI at 293 K of 1.053 g/cm^3^ and 79.5 kJ/mol; moreover, viscosity predictions dramatically improved, with a calculated value for HDI at 293 K of 2.8 mPas, much closer to the experimental value and representing a 10-fold improvement when compared with unparametrized GAFF results. As shown in Figure 6, the reparametrized force field was used to successfully reproduce the viscosity trends observed for functional and nonfunctional isocyanate trimers pure liquids and mixtures [98,99], meaning that it treats correctly the intermolecular interactions. This information was indeed used to investigate the effect of the intermolecular interaction of polyisocyanates on their viscosity, finding that the presence of NCO-Ring interactions is the main factor behind the viscosity rise in functional molecules [89]. 

The tremendous impact that a dedicated parametrization has on the predictive power of simulations also emerges from the recent work of Emelianova and Gor [100]. In their study, they reparametrized a TraPPE force field to accurately describe the isocyanate NCO group using *ab initio* calculations as reference data, and they used it to obtain accurate predictions of the vapor–liquid equilibrium curves of linear isocyanates. 

### 4.3. Coarse-Grained Models

Coarse-grained (CG) modeling [101,102], in which beads representing groups of atoms are employed, are required to simulate processes at larger time and length scales. There is a vast literature on the simulation of polyurethanes materials; however, in this paper, we wish to highlight those works that explicitly consider the network formation process involving isocyanates. 

The simpler coarse-graining step is to merge hydrogen atoms H with heavier ones A, so that –AH_x_ groups are treated as simple units, termed united atoms. While there are united atoms representations exist for the more popular force fields, such as for AMBER [103], they still suffer from the problems caused by a lack of parametrization for isocyanates, as proven in Ref. [100]. 

A coarser scale is obtained by replacing entire fragments or molecules with beads, as done by the MARTINI [104] or AWSEM-MD [105] force fields, developed for the study of large biomacromolecules. In such coarse-grained models, the modeling of chemical reactions, especially formation and degradation processes, is greatly simplified, since they can be represented as the formation and breaking of bonds between CG beads; moreover, while adding additional complexity of parametrization, coarse-graining reduces significantly the workload of simulation, because it reduces the amount of objects to model by about 90% [106]; thus, MARTINI force fields have been used to investigate the crosslinking formation process of PU networks [107] and the interaction between water and dangling chains at the network surface [108]. Such studies are fundamental in revealing how reaction conditions, such as solvent concentration, and mechanisms are strongly correlated with the predicted final crosslinked structure. In these works, MARTINI force field parameters have been obtained, starting from fully atomistic MD simulations using OPLS-AA [94] force field. It was noted that isocyanate properties, namely density, were poorly predicted by the CGFF. The same problem, that is, a large difference between the predicted and expected densities at 300 K, was also observed by the same group in the density of hexamethylene diisocyanate (HDI) [109], with a MARTINI-predicted density (at 300 K) of 0.89 g/cm^3^, that is a 15% underestimation with respect to the experimental value.

While these errors can still be neglected when the number of isocyanate molecules is much smaller than the other species, they will seriously affect predictions for systems with a large content of isocyanates, such as polyisocyanate networks and isocyanate melts. A correct treatment of the isocyanate moieties’ interactions between them and with the other species is fundamental to obtaining a reliable representation of the system’s dynamic properties and phases at any isocyanate concentration, especially if properties of the liquid phase properties or initial stages of network formation process are under investigation. As the inaccuracies of the MARTINI predictions originated from the use of a non-optimized force field, we expect that the availability of reliable force fields for the simulation of isocyanates and polyisocyanates can greatly improve the quality of the CG parameters and thus their simulation predictive power. 

Another coarse-grained method used for the mesoscale modeling and simulation of isocyanate systems is Dissipative Particle Dynamics (DPD) [110,111,112]. DPD provides some advantages compared to other CG methods. Firstly, DPD forces are always soft, i.e., there are no repulsive cores with diverging potentials, thereby allowing for much larger timesteps. In addition, DPD parameters might be directly related to Hildebrand solubility parameters [113], which can be obtained from all-atom MD simulations [114]. Incidentally, the quality of the final DPD parameters obtained in this way is strictly related to the accuracy of the underlying MD model. 

**Figure 7:**
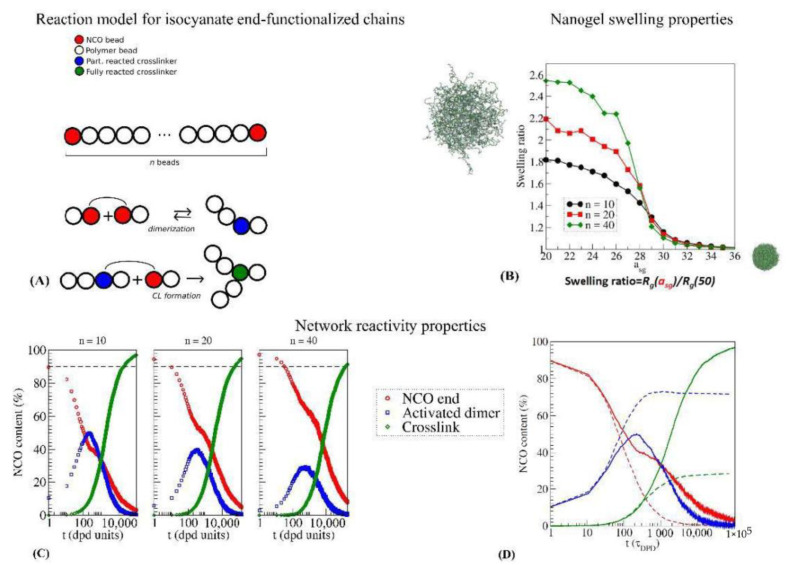
(**A**) representation of the reactive DPD model for isocyanate-end-functionalized chains [99,122]. (**B**) Swelling curves as a function of the solvent-gel interaction parameter *a_sg_* for isocyanate-end-crosslinked nanogels, for different chain lengths *n* of the precursor polymer melt. (**C**) Characterization of the crosslinking formation process as a function of precursor chain length. (**D**) Characterization of the crosslinking formation process for a reversible (full) and nonreversible (dashed) activated dimer reaction process. Color legend is the same for panels (**C**,**D**).

DPD has been used to study the formation and morphology of isocyanate-crosslinked networks at their free surface [115,116,117], polycarbonate-PU crosslinked materials [118], and polyethylene glycol hydrogels [119,120]. In the above-referred works, the precursor system is generally a mixture of linear polymers and aliphatic isocyanate trimers, i.e., already formed crosslinkers, and the urethane bond formation is simply modeled as a bond formation between the beads representing the isocyanate and alcohol groups. It has been shown that within this simple scheme, the reaction parameters do not influence the final structure and properties [121]. The formation of end-crosslinked nanogels from isocyanate-functionalized prepolymers has been studied using DPD [122]. In this case, the linking reaction is not the urethane bond formation, but the cyclotrimerization itself, which is modeled as a two-step reaction, as it is shown in Figure 7A, with the formation of an intermediate active NCO dimer. The simulated end-crosslinked nanogels demonstrated low content of residual isocyanates and good swelling properties (Figure 7B). Moreover, it was shown that the prepolymer chain length also influences the reaction process (Figure 7C), and that a reversible dimerization reaction is needed to achieve high crosslinking rates (Figure 7D).

An improvement to the DPD modeling physical properties of isocyanate-based material might be represented by the recently developed “slip-spring” method [123], where fictitious springs between chains to recover the effects of polymer entanglement, lost in DPD modeling, are introduced.

## 5. Outlook and Perspectives

Despite their long history, isocyanate molecules, especially aliphatic ones, are still continuously investigated. We have shown their role in defining the properties of crosslinked materials, as well as how they can be used in nanoscale-structured materials, from nanogels to functionalized surfaces. In this context, the simulation of the precursor melts and the network formation process is extremely helpful in revealing the complex relationship between microscopic structure and final properties; however, atomistic studies, especially using all-atom molecular dynamics models, have been generally limited by the availability of accurate force fields for isocyanates. The first steps have been already done in this direction [96,98]; however, a comprehensive force field capable of properly simulating aliphatic isocyanates and isocyanurates and their interactions with polyurethanes in all-atom MD has not been reached yet. Such a task is quite challenging, due to the rich interactions that even simple isocyanates are capable of Ref. [89], and the chemical complexity observed in polyurethanes. 

In our opinion, further efforts in parametrization should be directed towards the isocyanate group and isocyanurate ring interactions with urethane and urea groups, which are present in isocyanate-crosslinked materials, except pure polyisocyanate networks. This would allow us to obtain much better parameters for larger-scale CG simulations from atomistic ones, regardless of the method used.

Concerning aromatic isocyanates, to the best of our knowledge, there is no specifically developed parametrization yet. Compared to aliphatic ones, they are slightly more complex, as the interaction of the phenyl groups with both isocyanate and isocyanurate species should be taken into account. 

Recent advancements on modeling techniques such as reactive force fields and machine learning open new interesting directions for investigations. Reactive force fields, such as ReaxFF [124,125], and for which a variety of parametrization for organic molecules already exists, could be used to study the isocyanate reactivity e.g., in cyclotrimerization and urethane formation; however, the complex chemistry of the isocyanate groups imply the need of a very large initial parametrization set, and makes the parametrization strategy not obvious. Such work can be indeed made easier by using deep-learning assisted tools [126].

Machine learning tools are extremely powerful in providing atomistic force fields with *ab initio* accuracy [127]. In this sense, they are used to replace the parametrization process, in which the quantum mechanical forces are approximated by more or less accurate classical potentials, with an automated one. This allows for very accurate force fields that, when used in MD simulations, effectively combine the *ab initio* accuracy with the simulation of large time and length scales. In particular, Bayesian models [128] appear quite promising for organic liquids [129]. Concerning isocyanates, it should be possible to obtain a machine-learned force field for linear ones, with immediate applications in astrophysical problems, where simple isocyanates are of interest.

Conversely, the treatment of isocyanate trimers might be extremely challenging. In detail, the main obstacle in this sense is the complexity of the molecules and the presence of long-range noncovalent interactions, which translate into a very large number of training structures required to reach a good accuracy. Besides this, the transferability of these force fields should be tested with extreme care. With this said, it is clear that either reactive or machine-learned force field will dramatically expand our capabilities of simulating isocyanate-based systems; hence, the possibility of obtaining them should be regarded with extreme interest.

Regarding CG models, the development of machine learning and clustering algorithms allowed to automate the process of fragmentation of the graph of molecular bonds according to correlation of atomic displacements [130,131], or optimizing a general scoring function based on excluded volume and reproducing a number of thermodynamic quantities [132,133]. Such approaches might accelerate the optimization of CG parameters for isocyanate-based systems to enable their accurate mesoscale simulations. 

Finally, it is worth noting that, apart from reparametrization purposes, ML tools help to combine information from atomistic simulations with experimentally known data to predict macroscopic properties, thus avoiding the extreme burden of repetitive large-scale simulations, thereby helping to accelerate the development of isocyanate-based materials with the desired properties. In this fashion, it was possible to predict glass transition temperatures [134,135] and elastic properties [136,137] of isocyanate-containing polymers, based upon chemical molecular properties and chain mixtures simulations. 

## Figures and Tables

**Table 1:** Summary of the simulation techniques discussed in this review, along with their typical applications, advantages, and drawbacks.

Method	Applications	Advantages	Disadvantages
*Ab initio*	○ Calculations for isolated molecules, crystals, and small clusters. ○ Investigation of chemical reaction mechanisms. ○ Characterization of intra- and intermolecular interactions. ○ Adsorption properties.	○ High accuracy. ○ No need for external parameters. ○ Results can be used as reference data for force field parameterization at larger scales.	○ Computationally very intensive. ○ Limits in system size: up to hundreds of atoms.
Molecular dynamics	○ Prediction of physical and thermodynamic propertiesin condensed phases. ○ Study of energy, mass, momentum transport processes.	○ Much faster than *ab initio* methods. ○ Large-scale simulation of condensed phases. ○ Force fields are available for reactive studies.	○ Accuracy depends on the quality of parametrization. ○ The transferability of force field parameters needs testing.
Coarse-Grained Molecular Dynamics	○ Large-scale simulation of complex structures and their interfaces. ○ Study of network formation and degradation in crosslinked and phase-separated materials.	○ Simple implementation of network formation reactions. ○ Different levels of coarse-graining are available. ○ Very large length and time scales can be simulated.	○ Atomistic-scale details are lost. ○ Calculated CGMD interaction parameters affected by quality underlying MD.

## Data Availability

Data for the reproduction of the results reported in Figure 6 and Figure 7 can be provided by the corresponding author, upon reasonable request.
